# Bridging gaps in care: medical student home visits and their influence on radiation oncology patients

**DOI:** 10.1007/s00066-026-02508-1

**Published:** 2026-02-06

**Authors:** Jana Borgerding, Katrin Liethmann, David Krug, Christian Schulz, Jürgen Dunst, Amke Caliebe, Claudia Schmalz

**Affiliations:** 1 Department of Radiation Oncology, University Hospital Schleswig-Holstein, Campus Kiel, Arnold-Heller-Str. 3, 24105 Kiel, Germany; 2 Psycho-oncology, Center for Integrative Psychiatry ZiP gGmbH, Kiel, Germany; 3 Institut für Medizinische Informatik und Statistik, University Hospital Schleswig-Holstein, Campus Kiel, Kiel, Germany

**Keywords:** Supportive care, Medical education, Patient satisfaction, Patient experience, Healthcare transition

## Abstract

**Purpose:**

Patient care in radiation oncology is challenging. Interface problems can arise, particularly when transitioning from the inpatient to home environment. Inpatients’ perception of safety regarding the upcoming discharge and their satisfaction with care were addressed in the project.

**Methods:**

“Bridging the gaps” was an optional course for medical students in their 5th year. The study consisted of two arms—one with a home visit by medical students and the other without such a visit. Before discharge, inpatient radiation oncology patients were offered a home visit by medical students. A survey was conducted before (time point 1) and 3–5 days after discharge (time point 2) using questionnaires concerning satisfaction with care, current health status, and perception of safety. Outcome changes between time points 1 and 2 in both groups (with vs. without home visit) were compared.

**Results:**

A total of 60 patients were interviewed. Patients which received a home visit expressed improved perception of safety after discharge, whereas patients without a home visit showed decreased perception of safety (*p* = 0.024 for group-difference). Both groups showed a high level of satisfaction with care, which varied between the time points. In patients without a home visit, satisfaction decreased significantly after discharge, whereas satisfaction slightly increased in patients with a home visit (*p* = 0.001 for group-difference).

**Conclusion:**

Radiation oncology patients may benefit from home visits by increasing their perception of safety. Continuation and expansion of the project could strengthen the role of radiation oncology in the cross-sectoral care system.

**Supplementary Information:**

The online version of this article (10.1007/s00066-026-02508-1) contains supplementary material, which is available to authorized users.

## Introduction

Caring for patients in radiation oncology is challenging: throughout the whole treatment trajectory, there are several tasks to be completed. The North American National Cancer Institute (NCI) has highlighted some of the key challenges and emphasizes the importance of providing evidence-based, patient-centered services across the continuum of care. This includes timely and technically competent treatment, effective communication, shared decision-making, and cultural sensitivity, all aimed at improving clinical outcomes such as patient survival and health-related quality of life [1]. Radiotherapy, often in combination with chemotherapy or immunotherapy and usually administered as fractionated therapy with numerous treatment sessions, poses particular challenges. Marar et al. found that one third of treatment courses in radiation oncology with curative intent were associated with at least one hospital encounter, either during or shortly after treatment [2]. Before discharge, interface problems can arise. Many patients suffer from stress and a reduction in their general physical capacity and mental state during their oncological therapy, which can make it difficult to perform activities of daily living and participate in social life [3–6]. Recent studies on patients with breast, colorectal, and prostate cancer by Yang et al. and Hajdarevic et al. revealed feelings as anxiety, depression, and fear of recurrence only diminish over many months after the end of therapy, while shortly before discharge the desire to regain control can be observed [7, 8]. “Hospital discharge is a vulnerable stage in the patient pathway”, stated Waring et al. in 2013. Transitioning from the inpatient to the home environment and transferring to further therapies remains challenging for several tasks, e.g., coordination of supportive therapies between the hospital and the family doctor, knowledge transfer, and organizational support [9]. Dräger identified “the continuity of drug treatment and the lack of back-up systems after discharge” as relevant risks for patients’ safety after discharge in Germany [10]. Finally, treatment-related complications are emerging in oncology due to combination therapies, e.g., radiation and systemic treatments [11]. However, Beydoun et al. were able to show that improved conformity in radiotherapy not only reduces the risk of side effects but could also reduce problems associated with discharge [12]. Toxicities (e.g., from radiotherapy) usually reach their maximum around the end of therapy and have therefore not yet subsided by the time of discharge [13]. These problems in radio-oncological therapy represent a major challenge not only for the patients, but also for inexperienced practitioners.

Remarkably, studies have shown that home visits to vulnerable groups, such as elderly patients, significantly reduce emergency hospital admissions [14, 15]. Furthermore, it has been demonstrated that home visits positively influence patient satisfaction [16, 17]. To date, there have been no studies evaluating the specific situation of patients undergoing radiation therapy who are discharged from inpatient treatment and the effect of home visits for this patient group. Thus, it was the idea of this study, to introduce home visits as a possible strategy for reducing problems associated with discharge and lowering readmission rates.

For most patients in Germany, home visits to patients may only be carried out by doctors in private practice and not by hospital doctors. This German restriction that home visits may not be carried out by hospital doctors does not apply to medical students as part of their studies. In Germany, to prepare medical students as comprehensively as possible for the challenges of their future profession, there are plans to restructure teaching. Among other things, communicative skills in various dialogue situations (including home visits) are now explicitly mentioned as a central competence [18]. In a previous investigation, we were able to show that the field of radiotherapy can be particularly suitable for training interprofessionalism and communication skills in students [19]. Therefore, this research project was carried out in combination with a teaching project for medical students. As a first step in preparing this study, a preliminary 1‑month prestudy was conducted. This study on patients due to be discharged demonstrated the basic need for home visits and patients’ willingness to participate in the teaching project; further details are described in the methods section. Patients’ concerns and their perception of safety with regard to their upcoming discharge and their satisfaction with care were addressed in the “Bridging the gaps” teaching project.

Research questions:

- Are home visits by students to patients after discharge from inpatient therapy in radio-oncology feasible?
- What concerns do these patients report before discharge?
- Can home visits by medical students potentially influence patients’ concerns and satisfaction with their treatment?

## Methods

### Questionnaires

Three questionnaires were used in this study: one questionnaire (KI-21) concerning patients’ perception of safety and concerns towards the upcoming discharge was developed in the prestudy and two questionnaires were compiled with items from the European Organization for Research and Treatment of Cancer (EORTC) database [20]. In addition, data on the type of cancer, treatment, and secondary diagnoses were collected from the patient file.

#### Prestudy

In preparation for the prestudy in summer 2021 (ethics application D537/21), as a first step, a literature search was conducted (PubMed; “home visits”, “radiotherapy”, “radiooncology” “discharge”). Issue lists were developed. The questionnaire items were developed using an inductive, expert-based approach. Semi-structured interviews with open-ended questions were conducted with two radiation oncologists, one psychologist, and one social worker. A medical doctoral candidate and a psychologist conducted the interviews. Key points from the interviews were documented in written format and used to develop a first draft of the questionnaire items.

Subsequently, the preliminary questionnaire was sent to all experts for feedback and suggestions for revisions. This feedback was then incorporated into the final version of the questionnaire (final version of this questionnaire in the supplement). The development process followed established recommendations for inductive item generation and expert review in questionnaire development [21]. First, patients were asked whether they would like to participate in the prestudy and answer the questionnaire. After giving their consent, they were given the questionnaire and, if necessary, it was read aloud to them. Three areas were mentioned as possible causes for concern among patients: practical everyday tasks (e.g., cooking, cleaning, and making appointments with their practitioner), the absence or presence of a contact person, and physical complaints (e.g., pain, nausea/vomiting, weakness), persistence of complaints, or worsening of symptoms. As a next step, questions asking inpatients about possible fears and concerns regarding their discharge were formulated. Furthermore, respondents were asked about the availability of private and professional support options. Patients were also given the opportunity to add their own comments and concerns. In addition, willingness to participate in a home visit by students was queried.

During the 4‑week prestudy period, all patients were interviewed prior to their upcoming discharge.

#### KI-21

The KI-21 questionnaire was developed from the prestudy questionnaire and adapted based on feedback from participating patients. For the final version of the questionnaire, feedback was again sought from the expert group that developed the prestudy questionnaire. The final KI-21 (full questionnaire in the supplement) contained questions about sociodemographic data (gender, age, last profession) and living situation (living alone or with a partner/family, assisted living, or other). Patients were also asked whether they had to climb stairs in their home environment and what medical services were available to support them. Three questions addressed the patients’ perception of safety on a numeric rating scale from 10 = very safe to 0 = very unsafe with regard to discharge, care, and medical help in the home environment and further treatment planning. The last item asked whether the patient had any further concerns about discharge and if so, what they were. There were nine different options to choose from, several of which could be ticked, and a field where the patients could express individual worries.

#### EORTC IL-174 and IL-175

In the EORTC IL-174, medical doctors were rated in four areas: human qualities (e.g., empathy, willingness to listen, politeness, respect, and friendliness), time, thoroughness of physical examinations, and provision of information. In six items the organization of care was evaluated (e.g., information on planned examination dates, and support offers). One last item concerned the overall care in this clinic. For all items, a 5-point scale was provided (1 = poor, 2 = fair, 3 = good, 4 = very good, 5 = excellent). The second EORTC questionnaire (IL-175) contained questions about the patients’ current health. On a numeric rating scale from 1 = not at all to 4 = very much, the patients rated symptoms and problems of pain, weakness, tension, helplessness, and frustration. On the same scale, they also indicated how much their physical condition or medical treatment affected their socializing or activities with other people. In two final questions, patients were asked to rate their state of health and quality of life on a numeric rating scale from 1 = very poor to 7 = excellent.

### Study design and process

Based on the inclusion and exclusion criteria (Table 1) and the temporal feasibility of home visits, suitable patients were addressed: due to scheduling reasons, patients could only be included in the study by the students at selected times. The study included 60 participants, evenly divided between the two groups: with home visit and without home visit. Two to five days before discharge, patients were introduced to the project. After giving informed consent, patients were randomly assigned to a group. At each time point, the patients completed the same set of questionnaires: the KI-21, IL-174, and IL-175.

**Table 1:** Inclusion and exclusion criteria of potential study participants on the radiotherapy ward for possible inclusion in the pilot study with home visits by medical students

	Inclusion criteria	Exclusion criteria
All patients	Radiation oncology inpatients who are about to be discharged	Mental impairment leading to non-understanding of the consent or questionnaires
	Patients treated with radiation in palliative and curative intent	Patient is in the process of dying
		Insufficient understanding of the German language for communication and processing the questionnaires
Group *with* home visit	The patient’s home is located within a radius of < 100 km	The patient’s home is located outside a radius of > 100 km
		Questionable safety of students in the patient’s home environment

In the home visit group, patients had their first appointment with the students before discharge and completed the first set of questionnaires (A1). The medical students were prepared for the home visit through special and individualized training. During preparatory seminars, students learned about the support system for palliative patients in Germany. They also learned about the side effects of radiation therapy and how they are treated. Before the students met their assigned patients, a radiotherapy specialist explained the underlying oncological disease as well as relevant comorbidities and the treatment plan in advance. The typical course of disease and symptoms after discharge were discussed, which were to be monitored during the home visit. After discharge, the patients were visited by the students at home. The timing of the home visit was agreed upon jointly by the patient and the students (the specified period was 2–5 days after discharge). During the home visit, the patients were asked about their general condition based on a checklist and basic vital parameters were measured (see checklist in supplemental information). The patients completed the second set of questionnaires (A2) and could address any concerns or unanswered questions. For follow-up, the students met with one of the senior physicians, discussed the patients, and clarified questions. The home visits had no set duration. On average, a visit lasted about an hour. There were also no restrictions on whether or which relatives were allowed to attend the home visit. The students did not take on any organizational tasks, as patient follow-up care had already been arranged in advance.

The other group, without a home visit, completed the first set of questionnaires (B1) after giving consent and before being discharged. Two to five days after discharge, patients were contacted by telephone and completed the second set of questionnaires (B2).

Both groups completed the questionnaires by having the questions read aloud to them. They also had access to the questionnaires and were able to read the questions for themselves. The control group answered the second set of questionnaires by telephone.

Targeted advertising in specific social media groups and flyers distributed during lectures to the target group drew attention to the extracurricular teaching project. Interested students from the 5th year onwards were able to register and take part in an introductory information event. Inclusion criteria were successful completion of the first state medical examination and willingness to participate in the project.

### Sample size calculation

This study was a pilot study (ethics application D435/22). Therefore, the sample size calculation was geared towards a large effect. The primary outcome measure was the difference in inpatients’ perception of safety regarding the upcoming discharge between time points 1 and 2. Perception of safety was measured on an ordinal scale between 0 and 10 in the questionnaire. A nonparametric Wilcoxon rank sum test was used to compare the groups with and without a home visit. A large effect can be represented by Cohen’s d of 0.8, which corresponds to a Mann–Whitney estimator *P*(X < Y) of 0.7142 for the Wilcoxon rank sum test. For a significance level of 0.05 and a power of 0.80, this resulted in case numbers of *n* = 29 for the groups with and without home visits. The number of cases was calculated using the software program Bias for Windows, version 11.12 (https://www.bias-online.de).

Based on inclusion and exclusion criteria (Table 1), patients were recruited from April 2022 to February 2024.

### Statistical analysis

First, the differences between time point 1 and 2 were calculated:

$$X_{1}=A2-A1$$

$$X_{2}=B2-B1$$

A: Home visit group
B: Control group
1: time point 1 (before discharge)
2: time point 2 (after discharge)

Subsequently, the two groups were compared based on their differences (X_1_ and X_2_) using the Mann–Whitney U test for independent samples with a significance level of 5%. Moreover, 95% confidence levels were computed. This test was applied to all items and merged item groups in the questionnaires.

Fisher’s exact test was used to analyze any differences between categorical variables in the cohorts at time point 1.

Statistics were calculated with the program IBM SPSS Statistics version 29.0.0 (Armonk, NY, USA).

## Results

### Prestudy

During the 4‑week prestudy period, 57 inpatients were discharged, 31 of whom (54.38%) agreed to participate. Seven patients declined to participate, and 13 patients could not be interviewed (dementia, patients who could not be contacted sufficiently, language barrier). In 6 patients who had undergone brachytherapy alone, the survey did not seem appropriate. Of the 31 patients surveyed, 25 (80.65%) would agree to a home visit and would support such a project. The main arguments given for participation were the following: enabling students to learn something (76%), new medical questions (68%), help with medical problems (64%). Of the 31 patients, 11 (35.5%) reported having concerns about being discharged (multiple responses were possible). Three patients mentioned fear of loss of autonomy, two mentioned concerns about physical complaints, general fear of the future, and organizational difficulties, and one patient each mentioned the following concerns: practical matters, lack of a contact person, lack of joy in life, language difficulties, family problems, and concern that the illness would worsen.

### Patient collective

During the study, 250 inpatients were screened and 60 inpatients were asked to participate, all of whom agreed.

Both groups, with and without home visits, had a higher proportion of male participants (Table 2). The mean age differed slightly between the two groups, with the home visit group being older on average (70.6 years) compared to the control group (66.0 years). Most participants in both groups did not have an academic background, with 76.7% in the home visit group and 80% in the control group identifying as non-academics. The social environment of the patients’ homes showed no significant differences between the two groups. Approximately one-quarter of patients lived alone, while the majority lived in a family environment or with a partner (76.7% in the home visit group and 73.3% in the control group).

**Table 2:** Comparison of sociodemographic and clinical data of patients in the groups with vs. without a home visit at time point 1

	Group with home visit (*n* = 30)	Group without home visit (*n* = 30)
*Age*		
Mean age in years (standard deviation)	70.6 (± 8.87)	66.0 (± 7.51)
–	**Total (percentage)**	**Total (percentage)**
*Gender*		
Female	10 (33.3%)	14 (46.7%)
Male	20 (66.7%)	16 (53.3%)
*Last profession*		
Academic	7 (23.3%)	6 (20%)
Non-academic	23 (76.7%)	24 (80%)
*Climbing stairs at home necessary?*		
Yes	22 (73.3%)	19 (63.3%)
No	8 (26.7%)	11 (36.7%)
*Social environment at home*		
Living alone	7 (23.3%)	6 (20%)
Living with partner or family members	23 (76.7%)	22 (73.3%)
Assisted living	0	1 (3.3%)
Other	0	1 (3.3%)
*Therapy intention*		
Curative	18 (60%)	23 (76.7%)
Palliative	12 (40%)	7 (23.3%)
*Did the patient receive chemotherapy?*		
Yes	20 (66.7%)	22 (76.7%)
No	10 (33.3%)	8 (26.7%)
*Charlson Comorbidity Index (CCI)*		
0	15 (50%)	11 (36.7%)
1	5 (16.7%)	4 (13.3%)
2	6 (20%)	10 (33.3%)
3	3 (10%)	3 (10%)
4	1 (3.3%)	2 (6.7%)
*Location of the patient’s irradiation*		
Ear, nose, and throat area	14 (46.7%)	11 (36.7%)
Neurocranium	7 (23.3%)	4 (13.3%)
Rectum/anal/lower abdomen	3 (10%)	4 (13.3%)
Lung/mediastinum	3 (10%)	8 (26.7%)
Breast	0	1 (3.3%)
Female genitalia	0	2 (6.7%)
Bone metastases	3 (10%)	0

Distribution of treatment intention (curative vs. palliative) was 60 vs. 40% in the home visit group and 76.7 vs 23.3% in the control group. Chemotherapy was administered to 66.7% of patients in the home visit group compared to 76.7% in the control group. The CCI varied across both groups, with most patients having a CCI score of 0, indicating no significant comorbidities. A greater proportion of patients in the control group had a CCI > 0 (63.3% compared to 50% in the home visit group). The primary irradiation sites differed slightly between the groups. Both groups most frequently received irradiation to the ear, nose, and throat area (46.7% in the home visit group and 36.7% in the control group). Patients in the home visit group had irradiation to the neurocranium (23.3% vs. 13.3% in the control group) more often, while lung irradiation was more common in the control group (26.7% vs. 10% in the home visit group). For detailed data see Table 2.

### Variable analysis

The comparison of the groups at time point 1 (before discharge) showed no significant difference between the two groups for any question item (Mann–Whitney U‑test).

The Fisher exact test did not reveal any significant differences between male and female patients with regard to the significant results of the questionnaires.

### Feasibility of home visits

All appointed home visits could pe performed. A total of 31 home visits were made to 30 patients. One patient requested a second visit due to persistent physical problems during the first visit. A brief medical history was taken from all patients, questions were asked about radiation-related side effects, and vital signs were measured. In no case were findings requiring treatment identified. Afterwards, all findings were discussed with a senior radiation oncologist.

### Questionnaires

#### KI-21

**Fig. 1:**
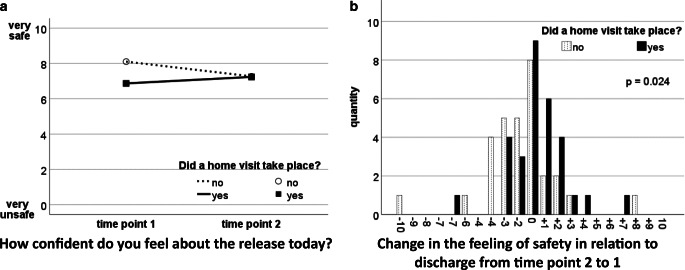
Change in responses to the question “How safe do you feel about the release today?”: average responses of the two groups over time (**a**) and change in the responses from time point 2 to 1 (**b**). Statistical evaluation of these changes (Mann–Whitney U test): *p* = 0.024

For the question on perception of safety concerning care and medical assistance in the home environment, the mean score was 8.0 across all patients on a scale of 0 = very unsafe to 10 = very safe, with no significant difference between groups (*p* = 0.235; Table 3). Perception of safety regarding further treatment planning yielded an average score of 7.1, with no significant group difference (*p* = 0.245). A difference between the groups was found in the perception of safety with regard to discharge (*p* = 0.024; Fig. 1). In the home visit group, the perception of safety slightly increased from 6.9 (before discharge) to 7.2 (after discharge), while a decrease from 8.1 to 7.3 was observed in the control group.

**Table 3:** Comparison of the results of the questionnaires of the two groups (with vs. without home visit). Indication of the respective mean values of the items before and after discharge and an analysis of the differences between the groups using the Mann–Whitney U test

	Mean value of group with home visit	Mean value of group without home visit	*p*-value of the difference comparison of the groups (Mann–Whitney‑U test)
**Self-designed questionnaire**			
*–*	***On a scale from 0 (= very uncertain)*** ***to 10 (= very confident)***		–
How confident do you feel about your release today?—before release	6.9 (± 2.75)	8.1 (± 2.14)	0.024
How confident do you feel about your release today?—after release	7.2 (± 2.37)	7.3 (± 2.85)	
How safe do you feel today with regard to your care and medical assistance at home?—before release	7.9 (± 2.75)	8.1 (± 1.91)	0.235
How safe do you feel today with regard to your care and medical assistance at home?—after release	7.7 (± 2.35)	8.3 (± 1.73)	
How confident do you feel today with regard to further treatment planning?—before release	6.7 (± 2.53)	7.5 (± 2.18)	0.245
How confident do you feel today with regard to further treatment planning?—after release	7.1 (± 2.62)	7.1 (± 2.26)	
*–*	***The sum of the number of stated worries: (max. 10 selectable)***		–
Is there anything that worries you about the release?—before release	1.7 (± 1.92)	1.3 (± 1.65)	0.900
Is there anything that worries you about the release?—after release	2.0 (± 2.02)	1.6 (± 1.76)	
**EORTC-IL-174**			
*Score evaluation*	***Score value of 50 possible points***		–
In this hospital, how would you rate doctors (10 questions on a scale from 1 [= poor] to 5 [= excellent])—before release	39.5 (± 8.43)	39.7 (± 6.06)	0.325
In this hospital, how would you rate doctors (10 questions on a scale from 1 [= poor] to 5 [= excellent])—after release	38.5 (± 8.88)	39.9 (± 7.23)	
*Score evaluation*	***Score value of 30 possible points***		–
In this hospital, how would you rate services and care organization (6 questions on a scale from 1 [= poor] to 5 [= excellent])—before release	21.3 (± 5.91)	21.1 (± 4.06)	0.650
In this hospital, how would you rate services and care organization (6 questions on a scale from 1 [= poor] to 5 [= excellent])—after release	20.4 (± 6.35)	20.9 (± 4.55)	
*In general,*	***On a scale from 1 (= poor) to 5 (= excellent)***		–
How would you rate the care you received in this hospital?—before release	3.9 (± 0.94)	4.2 (± 0.60)	0.001
How would you rate the care you received in this hospital?—after release	4.0 (± 0.93)	3.8 (± 0.74)	
**EORTC-IL-175**			
*During the past week:*	***On a scale from 1 (= not at all) to 4 (= very much)***		–
Have you had pain?—before release	2.1 (± 0.87)	1.7 (± 0.75)	0.039
Have you had pain?—after release	1.9 (± 0.96)	1.9 (± 0.80)	
Have you felt weak?—before release	2.4 (± 1.00)	2.1 (± 0.83)	0.089
Have you felt weak?—after release	2.5 (± 0.86)	2.6 (± 0.86)	
Did you feel tense?—before release	2.3 (± 1.04)	1.8 (± 0.83)	0.428
Did you feel tense?—after release	2.1 (± 0.96)	1.9 (± 1.01)	
Have you felt helpless?—before release	1.7 (± 0.91)	1.6 (± 0.68)	0.509
Have you felt helpless?—after release	1.8 (± 0.93)	1.5 (± 0.78)	
Have you felt frustrated?—before release	1.8 (± 0.96)	1.6 (± 0.68)	0.967
Have you felt frustrated?—after release	1.8 (± 0.89)	1.6 (± 0.77)	
Has your physical condition or medical treatment interfered with your family life?—before release	2.0 (± 1.13)	1.9 (± 0.86)	0.535
Has your physical condition or medical treatment interfered with your family life?—after release	2.0 (± 1.10)	2.0 (± 1.08)	
Has your physical condition or medical treatment interfered with your social activities?—before release	2.3 (± 1.18)	2.4 (± 0.93)	0.513
Has your physical condition or medical treatment interfered with your social activities?—after release	2.3 (± 1.16)	2.5 (± 1.14)	
*–*	***On a scale from 1 (= very poor) to 7 (= excellent)***		–
How would you rate your overall health during the past week?—before release	4.3 (± 1.52)	4.6 (± 0.97)	0.232
How would you rate your overall health during the past week?—after release	4.3 (± 1.46)	4.0 (± 1.13)	
How would you rate your overall quality of life during the past week?—before release	4.6 (± 1.63)	4.2 (± 1.33)	0.669
How would you rate your overall quality of life during the past week?—after release	4.4 (± 1.81)	3.8 (± 1.29)

Both groups reported a slight increase in the number of concerns postdischarge with no significant difference between groups: In the home visit group, from 1.7 before discharge to 2.0 after discharge, in the control group, from 1.3 to 1.6.

#### EORTC IL-174

On average, patients rated the treatment between 3.6 and 4.3 on a scale of 1 = poor to 5 = excellent. The highest average scores were given for human qualities, with scores of 4.2 before and 4.1 after discharge. Lower scores related to the time devoted to the patient and the provision of information, with average scores of 3.6 to 3.8.

The organization of care was also evaluated, with average values between 3.0 and 3.8. The ratings of the individual criteria were often lower after discharge than before (Table 3). There was no significant difference between the home visit group and the control group in either area, both in the assessment of the doctors and in the organization of care.

**Fig. 2:**
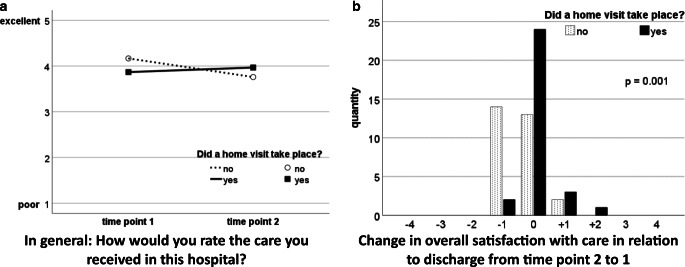
Change in responses to the question “In general: How would you rate the care you received in this hospital?”: average responses of the two groups over time (**a**) and change in the responses from time point 2 to 1 (**b**). Statistical evaluation of these changes (Mann–Whitney U test): *p* = 0.001

Concerning general assessment of care in hospital, the home visit group gave a score of 3.9 before discharge, which was stable at 4.0 after discharge. In the control group the rating was 4.2 before discharge and fell to 3.8 after discharge. The difference between the two groups proved to be significant (*p* = 0.001; Fig. 2).

#### EORTC IL-175

**Fig. 3:**
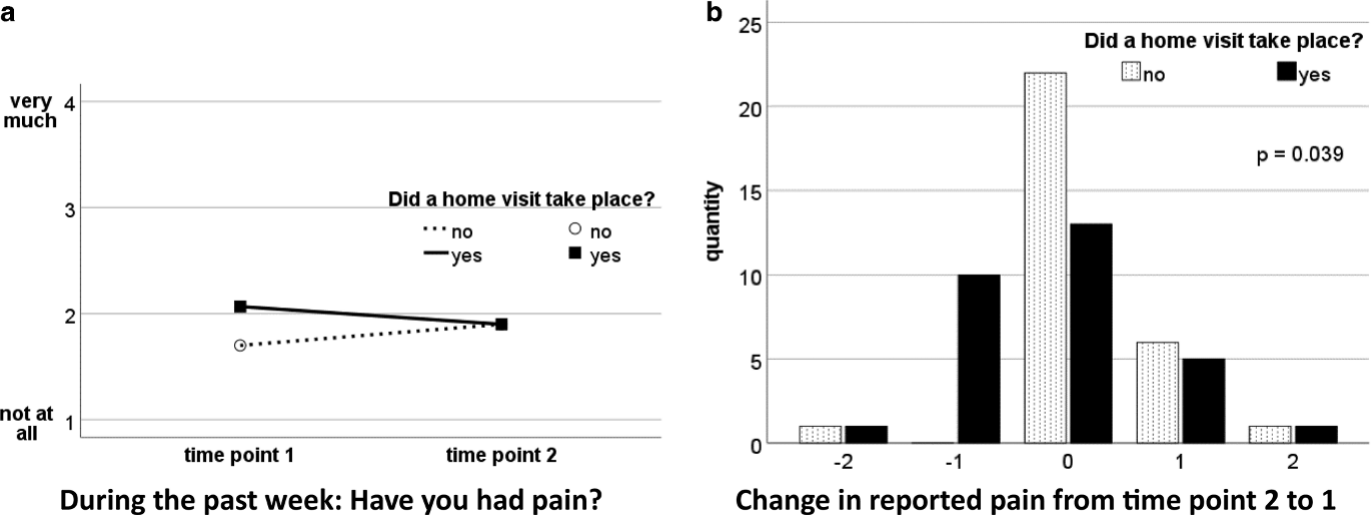
Change in responses to the question “During the past week: Have you had pain?”: average responses of the two groups over time (**a**) and change in the responses given from time point 2 to 1 (**b**). Statistical evaluation of these changes (Mann–Whitney U test): *p* = 0.039

Patients in both groups reported an impairment of their social life as a result of the therapy, with an average value of 2.4 on a scale of 0 = not at all to 4 = very much. The most frequently mentioned symptom was weakness, also with an average score of 2.4. Concerning the development of pain, both groups differed significantly: patients in the home visit group reported an average score of 2.1 before discharge and 1.9 after discharge. In the control group, the value was 1.7 before discharge and rose to 1.9 after discharge. The significance of *p* = 0.039 indicates a convergence of the pain scores in both groups (Fig. 3).

In the home visit group, general health during the past week was rated with an average score of 4.3 both before and after discharge on a scale of 1 = very poor to 7 = excellent. In the control group, this value was 4.6 before discharge and fell to 4.0 after discharge. The assessment of the general quality of life during the past week showed a value of 4.6 before discharge and 4.4 after discharge on a scale of 1 = very poor to 7 = excellent in the home visit group, while the control group gave a value of 4.2 before and 3.8 after discharge. There was no significant difference between the groups for either question.

## Discussion

Patient care in radiation oncology is challenging. On the one hand, side effects of therapy due to chemoradiation and radiation effects on healthy cells often result in the necessity of intensive care at the end of treatment. Discharge at the point of therapy completion is challenging [13]. On the other hand, limited healthcare resources (hospital bed capacity and the financial resources of insurance providers) prevent an unlimited extension of inpatient stays [22]. Even patients who are medically cleared for discharge may have many concerns and the transition into the home environment is particularly challenging for them. Patients with severe side effects are more likely to discontinue treatment early [23] or have to be re-admitted to the hospital unplanned [24].

Home visits as a part of primary care are widespread in Germany and play an important role in the healthcare system, as care problems and loneliness can be addressed [25]. Recently, Krause et al. stated that outpatient medical services in Germany appear to be more relevant than on average in Europe [26]. However, there are significant regional differences in the density of doctors in Germany, and the provision of care in rural and structurally weak areas is expected to become increasingly difficult in the future [27, 28]. The German Society of General Practice emphasizes the improvement of care for vulnerable groups [29].

This pilot study gives important evidence with regard to this question and many others. Due to the innovative concept, only a relatively small number of patients were included. Nevertheless, KI-21 showed a difference between the two groups with regard to the perception of safety after discharge: patients who received a home visit felt safer after discharge than before. Furthermore, it could be demonstrated that patients who received a home visit after discharge reported a higher level of satisfaction with care. To some extent, it seems to be a normal process that satisfaction with care increases in patients receiving special treatment as part of a study. In their extensive literature review on this question, Tantoy et al. came to the conclusion in 2021 that “little is known about the most appropriate patient satisfaction instrument to utilize and how to apply the domains of these existing instruments in patients who are enrolled in a clinical trial” [30]. The authors described a correlation between patient satisfaction and trial participation [30]. In the field of radiation oncology, Fabian et al. conducted a national survey in Germany which showed that patients were generally very satisfied with their treatment [31]. Recently, Baehr et al. demonstrated a close correlation between patient-centered care, the perception of safety, and satisfaction among radio-oncology patients [32]. The impact of personalized care, which was also part of the method used, plays a major role in this pilot study [33, 34, p. 159]. However, it seems remarkable that patients generally reported high level of satisfaction with care—after all, they had undergone strenuous cancer treatment [35]. The symptom-orientated questionnaire also showed an equalization of the groups. The two groups differed significantly in their pain reports: while pain decreased after discharge in the home visit group, it slightly rose after discharge in the control group. Presumably, the impact of personalized care and the associated placebo effect have an impact on pain, which means that a home visit could have a positive influence on pain reduction [33, 34, p. 159].

These significant results were achieved despite the fact that those carrying out the visits did not have a medical qualification. Furthermore, the home visits had no medical impact whatsoever; home visits were simply a visit. These results are in line with this. The reasons why patients felt satisfied or secure were not surveyed. In the future, it could be possible for home visits to be carried out by qualified nonmedical staff. Other optimizations (e.g., telephone visits) are also conceivable.

### Study limitations

The composition of the patient cohort is not representative of the general cancer distribution in Germany [36]: the patients surveyed were inpatients undergoing radiotherapy. Regarding the most frequently irradiated regions of our study participants, there is a more similar gender ratio between our data and the German cancer statistics [36].

Due to the design as an innovative pilot study, the number of patients was limited. Possibly, the differences between the cohorts have led to distortions. Patients in the home visit cohort may have had a different motivation to answer the questionnaires. The average age of the home visit group (70.6 years) is higher than that of the control group (66.0 years), which means that a poorer general condition at the start of treatment and less easily compensated side effects could be expected [37, 38]. Furthermore, home visits were scheduled 2–5 days after discharge. Using a standardized time period could improve the quality of the data collected. There may be a correlation between overall patient satisfaction and the time elapsed since discharge. It would be desirable to implement this in future studies.

Another important point is that the home visits were not standardized, e.g., the presence or absence of relatives or the length of the visits, which could have impacted the results [39].

The questionnaire K‑21 used in this study was self-designed and a validation study had not been conducted in advance. Patient satisfaction in radiation oncology has become a highly regarded topic; at the same time as this study was conducted, Pettegrew et al. developed a questionnaire (Radiation Oncology Patient Satisfaction [ROPS] questionnaire) on patient satisfaction in outpatient radiation oncology therapy [40]. The ROPS-Questionnaire could potentially complement our questionnaire in a future project.

Due to the exploratory nature of this pilot study, we did not make corrections for multiple testing. Therefore, the results should be verified in future studies with a larger sample size. By prespecifying the primary endpoint, the multiple testing problem for the research question was minimized [41, p. 230].

Unfortunately, it was not possible to analyze the benefits for the students as part of the pilot study, as the number of students participating was low. However, we did receive very good personal feedback from the participants. One possible explanation for the low willingness to participate in this extracurricular teaching project would be lack of time due to curricular events. Another explanation would be that radiotherapy has traditionally been taught only in the third year, when students are overloaded with new information [42]. Maybe, in their 5th year, when they were offered this project, the students knowledge about radiotherapy was not as current as hoped.

## Conclusion

This pilot study could show that home visits by students are feasible. The study indicates that patients may benefit from home visits concerning satisfaction with care and perception of safety. Repetition with randomization and blinded evaluation would be desirable for a future trial. A standardized examination of the benefits for the students would also be useful.

The concept tested in this pilot study is characterized by low costs, good feasibility, and the willingness of patients to participate in a home visit. Particularly in view of the increasing outpatient care in medicine [43] and a planned restructuring of medical studies in Germany [18], home visits by students could be an excellent solution to an interface problem. Transfer to other specialties beyond radiotherapy is also conceivable. However, intensive preparation and consultation with radiooncologists, who have the highest level of expertise in assessing and treating possible complications and atypical courses after inpatient radiation oncology treatment, should be maintained [44].

## Supplementary Information

ESM1: Supplementary material 1

ESM2: Supplementary material 2

ESM3: Supplementary material 3

ESM4: Supplementary material 4

ESM5: Supplementary material 5

ESM6: Supplementary material 6

ESM7: Supplementary material 7
