# Supplementary material for: Bridging gaps in care: medical student home visits and their influence on radiation oncology patients
Source: Strahlenther Onkol. 2026 Feb 6;202(7):722–33. doi: 10.1007/s00066-026-02508-1 (PMC13290831; doi:10.1007/s00066-026-02508-1)
Supplement: Supplementary file 1 — ESM1: Supplementary material 1 [file 66_2026_2508_MOESM1_ESM.pdf]

UKSH UNIVERSITÄTSKLINIKUM Schleswig-Holstein

CAU Christian-Albrechts-Universität zu Kiel
Medizinische Fakultät

Projekt „Weiter begleiten - Hausbesuche von Studierenden“

Fragebogen:

Wären Sie damit einverstanden, im Anschluss an die stationäre Therapie einen Hausbesuch zu erhalten?

- ☐ Ja
- ☐ Nein

Falls ja:

Was würden Sie sich davon erhoffen, dass Sie im Anschluss an die stationäre Therapie einen Hausbesuch erhalten?

……………………………………………………………………………………………

……………………………………………………………………………………………

……………………………………………………………………………………………

……………………………………………………………………………………………

Falls nein:

Wären Sie bereit, uns die Gründe zu nennen, warum Sie nicht mit einem Hausbesuch einverstanden sind?

……………………………………………………………………………………………

……………………………………………………………………………………………

……………………………………………………………………………………………

……………………………………………………………………………………………

Gibt es irgendetwas, was Ihnen Sorgen bereitet in Bezug auf die Entlassung?

- ☐ Ja
- ☐ Nein

Falls ja:

Was bereitet Ihnen Sorgen:

- ☐ Lebenspraktische Dinge: wer kauft ein, wer kocht, wer macht die Wäsche, wer saugt Staub?
- ☐ Fehlen eines Ansprechpartners: mit wem kann ich sprechen? An wen kann ich mich wenden?
- ☐ Körperliche Beschwerden (z. B. Schmerzen, Übelkeit/ Erbrechen, Schwäche), Anhalten der Beschwerden oder Verschlimmerung

Wieviel Unterstützung durch private / familiäre/ freundschaftliche Kontakte haben Sie im häuslichen Umfeld?

- ☐ Viel Unterstützung
- ☐ Gute Unterstützung
- ☐ Mittel
- ☐ wenn es notwendig ist, wird jemand da sein
- ☐ keine Unterstützung

Welche medizinische Unterstützung / Kontakte haben Sie im häuslichen Umfeld?

- ☐ Durch meine HausärztIn / FachärztIn
- ☐ Durch einen Pflegedienst (bereits bekannt)
- ☐ Durch einen Pflegedienst (neu)
- ☐ Durch einen Palliativdienst (bereits bekannt)
- ☐ Durch einen Palliativdienst (neu)
- ☐ Durch die Hospizinitiative
- ☐ Andere: …………………………………………………………………………

Bitte geben Sie an, in welcher Körperregion Sie bestrahlt werden:

- ☐ Gehirn
- ☐ Kopf- und Halsregion
- ☐ Oberkörper / Lunge
- ☐ Bauch / Becken
- ☐ Extremitäten

Vielen Dank!
