# Supplementary material for: Bridging gaps in care: medical student home visits and their influence on radiation oncology patients
Source: Strahlenther Onkol. 2026 Feb 6;202(7):722–33. doi: 10.1007/s00066-026-02508-1 (PMC13290831; doi:10.1007/s00066-026-02508-1)
Supplement: Supplementary file 2 — ESM2: Supplementary material 2 [file 66_2026_2508_MOESM2_ESM.pdf]

Datum: Uhrzeit-Ankunft:
Patient: Uhrzeit-Abfahrt:
Studierender 1:
Studierender 2:
Hausarzt (mit Telefonnummer):

Messungen

Puls (b/min)	Blutdruck (mmHg)	SpO2 (%)	Temperatur	BZ (bei Diabetikern)

Allgemeinzustand:

(Frage nach Wohlbefinden, Möglichkeit der Selbstversorgung- wie eigenständige Toilette, Arztbesuche, Einkauf, Evtl. häusliche Unterstützung-wenn ja durch wen? Allgemeines Zurechtkommen im häuslichen Umfeld?)

Medikation:

(Vor allem die Grundlegende Standardmedikation+ Bedarfsmedikation)

Symptome:

(Welche? Verbesserung/ Verschlechterung, Stärke, möglicherweise UAW der Medikation?)

	Verbesserung	Verschlechterung	Stärke/ Häufigkeit	Medikation
Übelkeit/ Erbrechen				
Durchfall				
Schmerzen				
o Lokalisation				
Müdigkeit/ Fatigue				
Sonstige				

Wundprotokoll (bei Radiodermatitis)

Größe:_________________

Tiefe:_________________

Aussehen

- o Rötung
- o Epitheliolyse
- o Superinfektion
- o Blasig
- o Nekrotisch

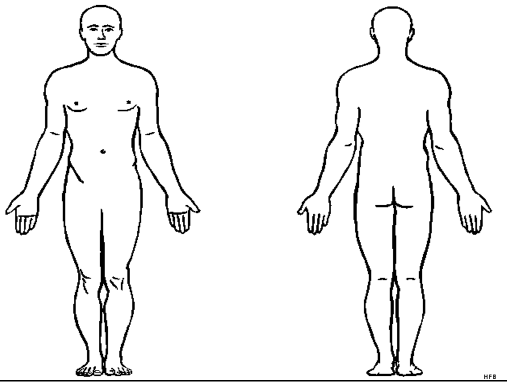

Notizen/ sonstige Anmerkungen:
