# Supplementary material for: Bridging gaps in care: medical student home visits and their influence on radiation oncology patients
Source: Strahlenther Onkol. 2026 Feb 6;202(7):722–33. doi: 10.1007/s00066-026-02508-1 (PMC13290831; doi:10.1007/s00066-026-02508-1)
Supplement: Supplementary file 3 — ESM3: Supplementary material 3 [file 66_2026_2508_MOESM3_ESM.docx]

**Measurements**

| **pulse (b/min)** | **blood pressure (mmHg)** | **SpO2 (%)** | **temperature** | **BS (diabetics)** |
| --- | --- | --- | --- | --- |
|  |  |  |  |  |

**General condition:**

(Ask about well-being, ability to take care of oneself—such as using the toilet independently, visiting the doctor, shopping, possibly receiving domestic support—if so, from whom? General ability to cope in the home environment?)

|  |
| --- |

**Medication:**

(Above all, basic standard medication + medication as needed)

|  |
| --- |

**Symptoms:**

(Which ones? Improvement/deterioration, severity, possible adverse reactions to medication?)

|  | **Improvement** | **Deterioration** | **Severity/ Frequency** | **Medication** |
| --- | --- | --- | --- | --- |
| Nausea/ Vomiting |  |  |  |  |
| Diarrheal |  |  |  |  |
| Pain |  |  |  |  |
| - Localization |  | | | |
| Tiredness/ Fatigue |  |  |  |  |
| Other |  | | | |


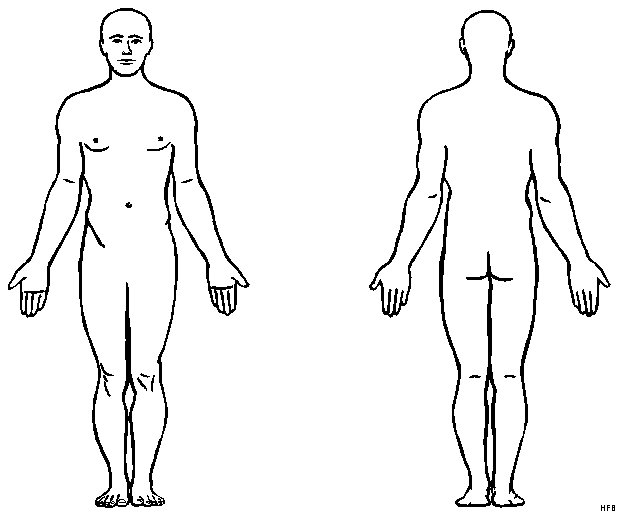
**Wound report (in cases of radiodermatitis)**

Size:________________

Depth:_________________

Appearance

- Redness
- Epitheliolysis
- Superinfektion
- Blistered
- Necrotic

**Notes/ other comments:**

|  |
| --- |
