# Supplementary material for: Bridging gaps in care: medical student home visits and their influence on radiation oncology patients
Source: Strahlenther Onkol. 2026 Feb 6;202(7):722–33. doi: 10.1007/s00066-026-02508-1 (PMC13290831; doi:10.1007/s00066-026-02508-1)
Supplement: Supplementary file 4 — ESM4: Supplementary material 4 [file 66_2026_2508_MOESM4_ESM.pdf]

EORTC IL174

Wir interessieren uns für Ihre zuletzt gemachten Erfahrungen mit der Betreuung, die Sie in diesem Krankenhaus oder in dieser Fachambulanz erhalten haben. Beantworten Sie bitte alle Fragen selbst und umkreisen Sie dafür die Zahl, die am besten auf Sie zutrifft. Es gibt keine „richtigen“ oder „falschen“ Antworten. Ihre Angaben werden streng vertraulich behandelt.

Bitte geben Sie an, welche Krankenhausabteilung oder Ambulanz Sie bewerten (bitte nur eine auswählen):

Bei stationärer Betreuung: □

Bei ambulanter Betreuung: □

Wie würden Sie die Ärzte und Ärztinnen in diesem Krankenhaus bezogen auf folgende Kriterien bewerten:

	Schlecht	Mittel-mäßig	Gut	Sehr gut	Ausge-zeichnet	Nicht zu-treffend
1. Die Beachtung, die sie Ihren körperlichen Symptomen schenkten?	1	2	3	4	5	n.z.
2. Die Sorgfalt bei der Behandlung Ihrer körperlichen Symptome?	1	2	3	4	5	n.z.
3. Das Interesse, das sie Ihnen als Person entgegenbrachten?	1	2	3	4	5	
4. Die Zuwendung und Unterstützung, die sie Ihnen gaben?	1	2	3	4	5	
5. Die Häufigkeit der Visiten / Arztgespräche?	1	2	3	4	5	
6. Die Zeit, die sie Ihnen gewidmet haben?	1	2	3	4	5	
7. Die Auskünfte, die sie Ihnen zu Ihrer Versorgung und Behandlung gaben?	1	2	3	4	5	n.z.
8. Ihrer Bereitschaft, Ihnen zuzuhören?	1	2	3	4	5	
9. Ihren menschlichen Qualitäten (Höflichkeit, Respekt, Einfühlungsvermögen, Freundlichkeit, Geduld, usw.)?	1	2	3	4	5	
10. Der Ihnen gewidmeten Zeit bei Visiten/ärztlicher Beratung?	1	2	3	4	5	

Bitte auf der nächsten Seite fortsetzen

**Wie würden Sie <u>Dienstleistungen und die Organisation der Betreuung</u> in diesem Krankenhaus bezogen auf folgende Kriterien bewerten:**	**Schlecht**	**Mittel-mäßig**	**Gut**	**Sehr gut**	**Ausge-zeichnet**	**Nicht zu-treffend**
11. Der Austausch von Informationen zwischen den unterschiedlichen Berufsgruppen (Ärzte, Pflegekräfte, Physiotherapeuten, Psychologen usw.), die in Ihre Behandlung eingebunden sind?	1	2	3	4	5	
12. Der Austausch von Informationen mit anderen Gesundheitsdienstleistern in Ihrer Region (Hausarzt, häusliche Pflege, Pflegeheim, Sozialdienste usw.)?	1	2	3	4	5	n.z.
13. Die Informationen zur Planung Ihrer Untersuchungs- und Behandlungstermine?	1	2	3	4	5	
14. Die Informationen zu verfügbaren Unterstützungsangeboten (sozialer oder psychologischer Dienst, Physiotherapie, Ernährungsberatung, Selbsthilfegruppe usw.)?	1	2	3	4	5	
15. Die erteilten Auskünfte darüber, was Sie nach Ihrem Krankenhaustermin tun sollten und was nicht?	1	2	3	4	5	
16. Die Auskünfte, an wen Sie sich wenden können, wenn Sie nach Ihrem Krankenhaustermin besorgt sind?	1	2	3	4	5	
**Im Allgemeinen:**						
17. Wie würden Sie die Versorgung bewerten, die Sie in diesem Krankenhaus erhalten haben?	1	2	3	4	5	

© Copyright 2022 EORTC Quality of Life Group. All rights reserved.
