# Supplementary material for: Bridging gaps in care: medical student home visits and their influence on radiation oncology patients
Source: Strahlenther Onkol. 2026 Feb 6;202(7):722–33. doi: 10.1007/s00066-026-02508-1 (PMC13290831; doi:10.1007/s00066-026-02508-1)
Supplement: Supplementary file 5 — ESM5: Supplementary material 5 [file 66_2026_2508_MOESM5_ESM.pdf]

EORTC IL175

Patienten berichten manchmal die folgenden Symptome oder Probleme. Bitte geben Sie an, in welchem Ausmaß Sie diese Symptome oder Probleme während der letzten Woche erlebt haben. Markieren Sie bitte die Zahl, die am besten auf Sie zutrifft.

**Während der letzten Woche:**	**Überhaupt nicht**	**Wenig**	**Ziemlich**	**Sehr**
1. Hatten Sie Schmerzen?	1	2	3	4
2. Fühlten Sie sich schwach?	1	2	3	4
3. Fühlten Sie sich angespannt?	1	2	3	4
4. Haben Sie sich hilflos gefühlt?	1	2	3	4
5. Fühlten Sie sich frustriert?	1	2	3	4
6. Hat Ihr körperlicher Zustand oder Ihre medizinische Behandlung Ihr Familienleben beeinträchtigt?	1	2	3	4
7. Hat Ihr körperlicher Zustand oder Ihre medizinische Behandlung Ihr Zusammensein oder Ihre gemeinsamen Unternehmungen mit anderen Menschen beeinträchtigt?	1	2	3	4

Bitte kreisen Sie bei den folgenden Fragen die Zahl zwischen 1 und 7 ein, die am besten auf Sie zutrifft:

8. Wie würden Sie insgesamt Ihren Gesundheitszustand während der letzten Woche einschätzen?

1	2	3	4	5	6	7
sehr schlecht						ausgezeichnet

9. Wie würden Sie insgesamt Ihre Lebensqualität während der letzten Woche einschätzen?

1	2	3	4	5	6	7
sehr schlecht						ausgezeichnet

© Copyright 2022 EORTC Quality of Life Group. All rights reserved.
