# Supplementary material for: Bridging gaps in care: medical student home visits and their influence on radiation oncology patients
Source: Strahlenther Onkol. 2026 Feb 6;202(7):722–33. doi: 10.1007/s00066-026-02508-1 (PMC13290831; doi:10.1007/s00066-026-02508-1)
Supplement: Supplementary file 6 — ESM6: Supplementary material 6 [file 66_2026_2508_MOESM6_ESM.docx]

**Questionnaire „Bridging the gaps“**

*First, a few questions about you*

Your gender is

- male
- female
- diverse

How old are your? .…… years

Your last job is/was

…………………………………………………………………………

Do you need to be able to climb stairs in your apartment?

- yes
- no

What is your social environment like at home?

- living alone
- Living with partner or family members
- assisted livingBetreutes Wohnen
- differently, namely………………………………………………………..

**How safe do you feel today …?**

… with regard to your discharge today?

**10 9 8 7 6 5 4 3 2 1 0**

Very safe very insecure

… with regard to your care and medical assistance in your home environment?

**10 9 8 7 6 5 4 3 2 1 0**

Very safe very insecure

… with regard to further treatment planning?

**10 9 8 7 6 5 4 3 2 1 0**

Very safe very insecure

**Is there anything that worries you about the release?**

- yes
- no

***If yes:***

**What worries you:**

- Practical matters: who does the shopping, who cooks, who does the laundry, who vacuums?
- No contact person: Who can I talk to? Who can I turn to?
- Physical symptoms (e.g., pain, nausea/vomiting, weakness), persistence of symptoms, or worsening of symptoms
- Lack of independence
- General fear of the future
- Lack of joy in life
- Language difficulties
- Worry that the disease will worsen
- Family problems
- Something else entirely, namely…………………………………………..

**After discharge, you can expect support from the following medical services**

- From your family doctor/specialist
- From a nursing service
- From a palliative care service
- From a hospice service
- Other: ……………………………………………………………………

Thanks very much!
