# Supplementary material for: Bridging gaps in care: medical student home visits and their influence on radiation oncology patients
Source: Strahlenther Onkol. 2026 Feb 6;202(7):722–33. doi: 10.1007/s00066-026-02508-1 (PMC13290831; doi:10.1007/s00066-026-02508-1)
Supplement: Supplementary file 7 — ESM7: Supplementary material 7 [file 66_2026_2508_MOESM7_ESM.pdf]

UKSH UNIVERSITÄTSKLINIKUM Schleswig-Holstein

CAU Christian-Albrechts-Universität zu Kiel
Medizinische Fakultät

Fragebogen „Weiter begleiten“

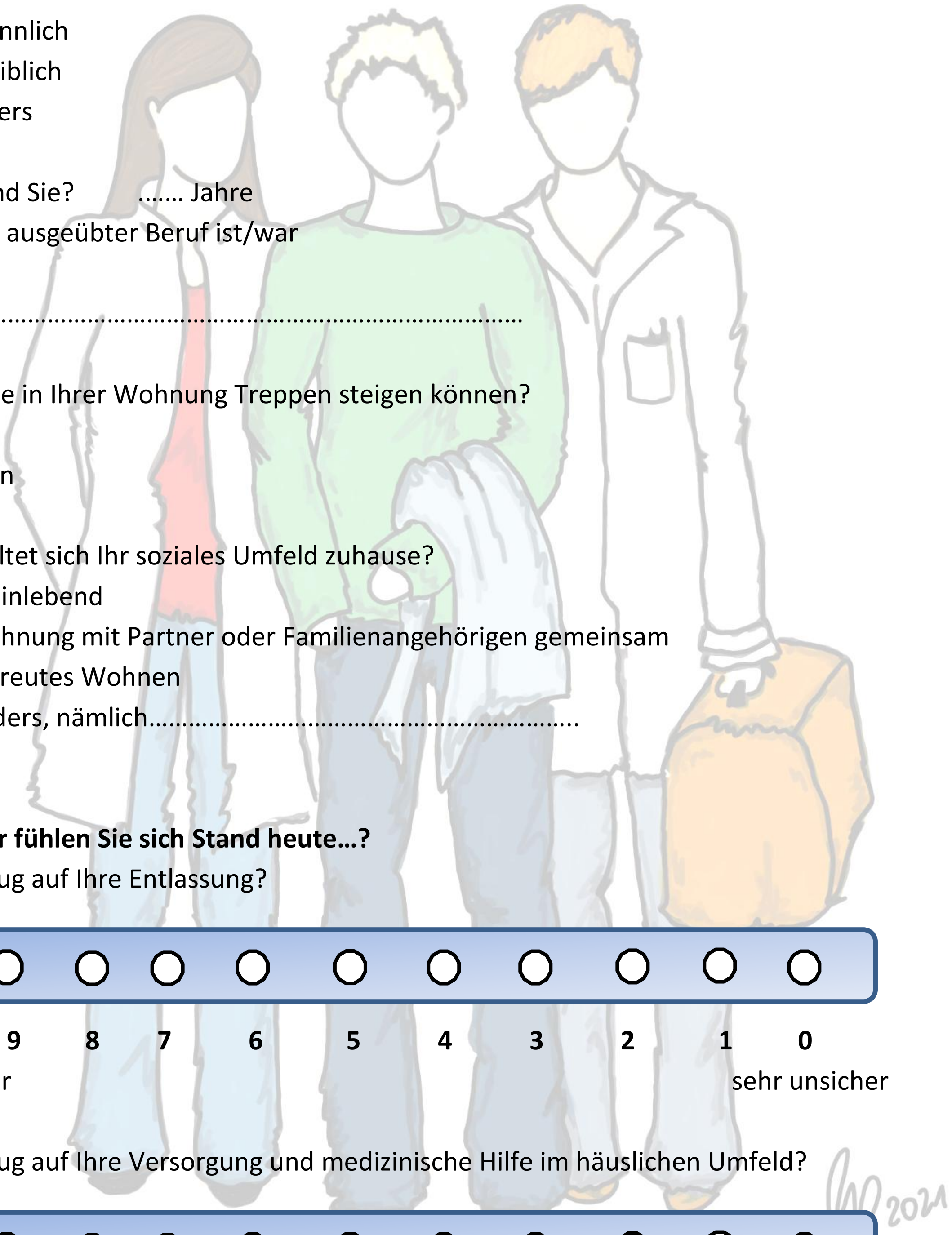

Zunächst einige Fragen zu Ihnen

Ihr Geschlecht ist

- ☐ Männlich
- ☐ Weiblich
- ☐ Divers

Wie alt sind Sie? ……. Jahre

Ihr zuletzt ausgeübter Beruf ist/war

………………………………………………………………………………

Müssen Sie in Ihrer Wohnung Treppen steigen können?

- ☐ Ja
- ☐ Nein

Wie gestaltet sich Ihr soziales Umfeld zuhause?

- ☐ alleinlebend
- ☐ Wohnung mit Partner oder Familienangehörigen gemeinsam
- ☐ Betreutes Wohnen
- ☐ Anders, nämlich………………………………………………………..

Wie sicher fühlen Sie sich Stand heute…?

… mit Bezug auf Ihre Entlassung?

○ ○ ○ ○ ○ ○ ○ ○ ○ ○ ○

10 9 8 7 6 5 4 3 2 1 0

Sehr sicher sehr unsicher

… mit Bezug auf Ihre Versorgung und medizinische Hilfe im häuslichen Umfeld?

○ ○ ○ ○ ○ ○ ○ ○ ○ ○ ○

10 9 8 7 6 5 4 3 2 1 0

Sehr sicher sehr unsicher

… mit Bezug auf die weitere Therapieplanung?

○	○	○	○	○	○	○	○	○	○	○
**10**	**9**	**8**	**7**	**6**	**5**	**4**	**3**	**2**	**1**	**0**
Sehr sicher										sehr unsicher

Gibt es irgendetwas, was Ihnen Sorgen bereitet in Bezug auf die Entlassung?

- ☐ Ja
- ☐ Nein

Falls ja:
Was bereitet Ihnen Sorgen:

- ☐ Lebenspraktische Dinge: wer kauft ein, wer kocht, wer macht die Wäsche, wer saugt Staub?
- ☐ Fehlen eines Ansprechpartners: mit wem kann ich sprechen? An wen kann ich mich wenden?
- ☐ Körperliche Beschwerden (z. B. Schmerzen, Übelkeit/ Erbrechen, Schwäche), Anhalten der Beschwerden oder Verschlimmerung
- ☐ Fehlende Selbstständigkeit
- ☐ Generelle Zukunftsangst
- ☐ Fehlende Lebensfreude
- ☐ Sprachschwierigkeiten
- ☐ Sorge, dass sich die Krankheit verschlimmert
- ☐ Familiäre Probleme
- ☐ Etwas ganz anderes, nämlich…………………………………………….

Nach der Entlassung erwarten Sie eine Unterstützung durch folgende medizinische Angebote

- ☐ Durch Ihre HausärztIn / FachärztIn
- ☐ Durch einen Pflegedienst
- ☐ Durch einen Palliativdienst
- ☐ Durch einen Hospizdienst
- ☐ Andere: ……………………………………………………………………

Vielen Dank!
